# Exploring the treatment of liver cancer with Gehua Hugan Gao based on bioinformatics, network pharmacology, and molecular docking

**DOI:** 10.1097/MD.0000000000050231

**Published:** 2026-08-28

**Authors:** Guangze Ye, Zhu Yang, Bing Yang, Dalin Chen, Dan Li, Hongting Yan, Hanbo Qiu, Fengxi Long, Wenyu Wu, Dongxin Tang

**Affiliations:** a Guizhou University of Traditional Chinese Medicine, Guiyang, Guizhou, China; b Medical Translational Engineering Research Center for Prevention and Treatment of Oncology of Traditional Chinese Medicine and Ethnomedicine in Higher Education Institutions, Guiyang, Guizhou, China; c TCM Tumor Inheritance and Scientific and Technological Innovation Talent Base of Guizhou, Guiyang, Guizhou, China; d Guizhou Hospital of the First Affiliated Hospital, Sun Yat-sen University, Guiyang, Guizhou, China; e First Affiliated Hospital of Guizhou University of Traditional Chinese Medicine, Guiyang, Guizhou, China.

**Keywords:** Gehua Hugan Gao, liver cancer, molecular docking, network pharmacology

## Abstract

Gehua Hugan Gao (GHHGG) is a traditional Chinese medicine paste that is chiefly used to treat liver cancer. However, the potential impact of GHHGG on liver cancer remains unclear. We explored how GHHGG treats liver cancer using bioinformatics, network pharmacology, and molecular docking. Network pharmacology included GHHGG active ingredients, predicted targets, predicted targets for liver cancer, and differential gene collection. A protein-protein interaction network was constructed using the Search Tool for the Retrieval of Interacting Genes/Proteins database, and crucial targets were ranked according to their degree values. Gene Ontology and Kyoto Encyclopedia of Genes and Genomes analyses of liver cancer targets were followed by survival, differential analysis, and molecular docking. Venn diagrams show 123 predicted GHHGG targets for the treatment of hepatocellular carcinoma (HCC). Enrichment analysis showed that GHHGG treats HCC through multiple targets and pathways. We also found that estrogen receptor 1, cytochrome P450 3A4, cyclin-dependent kinase 4, type IIA topoisomerase, aurora kinase A, and cyclin E1 targets were closely associated with HCC development through survival and differential analyses. Molecular docking confirmed GHHGG’s strong affinity for liver cancer targets. This study helps us understand GHHGG ingredients and targets for liver cancer treatment. To a certain extent, the molecular mechanism of GHHGG in the treatment of liver cancer has been elucidated, thus providing a theoretical basis.

## 1. Introduction

Liver cancer, also known as primary liver cancer, is the sixth most common cancer and the third leading cause of cancer deaths, according to the 2020 Global Cancer Statistics.^[1]^ One study predicted a 55 percent increase in new liver cancer diagnoses and fatalities by 2040.^[2]^ Hepatocellular carcinoma (HCC) accounts for about 90% of liver cancer cases.^[3]^ Despite continuous progress in treating liver cancer, drug development has been slow, and most drugs are ineffective in treating patients. Therefore, it has become an urgent task to develop alternative medicines with better efficacy and lower toxicity to improve the survival rate of liver cancer patients. Owing to their low toxicity and multifactorial and multi-pathway targeting, Chinese herbal medicines have long been used in clinical practice, and they have great potential for the development of new anti-liver cancer natural medicines.^[4]^ Gehua Hugan Gao (GHHGG), based on the theory of “Drinking Injury” and combining years of clinical experience in cancer prevention and treatment, Prof Yang Zhu, a Qihuang scholar in China, has added a series of traditional Chinese medicines (TCMs) that are commonly used in the treatment of tumors based on Gehua Jiecheng Decoction. Ointment is a dosage form of TCM, consisting of many kinds of TCMs that are easy to take, taste good, and comply well with patients. GHHGG has been used for many years in the First Affiliated Hospital of Guizhou University of TCM as an auxiliary treatment for liver cancer, and its main component, Gehua Jiecheng Decoction, has been proven to have an inhibitory effect on HCC.^[5]^ The impact of GHHGG on liver cancer regulation lacks a clear understanding of its mechanism, as no study has been conducted on this subject. TCM’s “poly-component, poly-target, poly-pathway” approach can effectively treat complex diseases. TCM-disease interactions require new approaches. Network pharmacology reveals TCM formula mechanisms.^[6]^ The development and application of network pharmacology have facilitated research on the safety, effectiveness, and mechanisms of action of Chinese medicines.^[7]^ If we go through the literature, we will find many studies reporting the use of network pharmacology to treat cancer with Chinese herbal medicine. Jiao et al found that *Scutellaria baicalensis* has a multi-component, multi-target effect on breast cancer treatment. AKT serine/threonine kinase 1, which targets Huanglian, was the most effective compound.^[7]^ Bazhen Tang can inhibit colorectal cancer progression and boost anticancer immune function in mice. This was verified through network pharmacology, molecular docking, and experiments, which showed that Bazhen Tang can regulate signaling pathways such as phosphatidylinositol 3-kinase–protein kinase B, P53, and Vascular Endothelial Growth Factor Receptor, and reverse abnormal gene expression of PI3K, Vascular endothelial Growth Factor Receptor, and TP53.^[8]^ A network pharmacology study of the mechanisms of the Jiedu Qingjin formulation for treating non-small cell lung cancer suggested that pathways related to epithelial-mesenchymal transition, inflammation, immunity, and angiogenesis are involved in the progression of non-small cell lung cancer.^[9]^ Sijunzi Tang induced apoptosis and autophagy in colorectal cancer cells via the PI3K/Akt/mechanistic target of rapamycin pathway, as demonstrated by Shang et al using network pharmacology and experimental validation.^[10]^ To our knowledge, this is the first study to explore GHHGG’s mechanism of action for the treatment of liver cancer. Here, we studied the active compounds and targets of GHHGG. It was supplemented with survival analysis, differential gene analysis, and molecular docking studies to validate the results. This study sheds new light on GHHGG’s anti-liver cancer activity and can help accelerate drug discovery. Further laboratory experiments are required to analyze its pharmacological potential.

## 2. Material and methods

**Figure 1.**
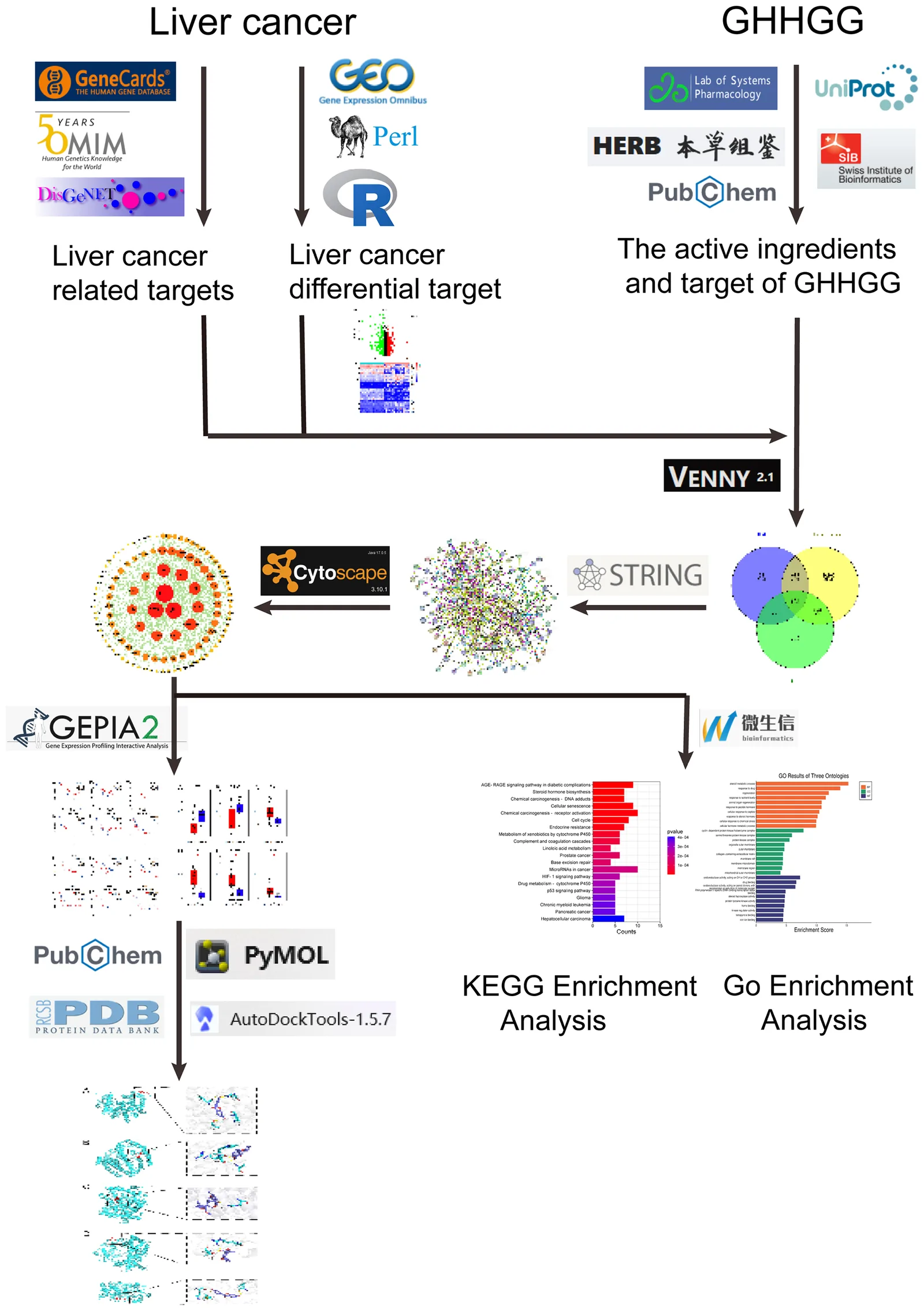
Flowchart of this study. GEO = Gene Expression Omnibus, GHHGG = Gehua Hugan Gao.

The overall analytical workflow of this study is summarized in Figure 1.

### 2.1. Acquisition of target genes for liver cancer

To search for genes related to liver cancer, we used MeSH (https://www.ncbi.nlm.nih.gov/mesh/) to find free words with the keyword “liver cancer.” These free words were then searched in the GeneCards (http://www.genecards.org/), DisGeNET (https://www.disgenet.org/), and Online Mendelian Inheritance in Man (OMIM; http://omim.org/) databases to identify disease-related genes. Next, we searched for disease-related genes in each of the 3 databases and removed duplicates to obtain a list of potential targets related to liver cancer.

### 2.2. Screening for HCC differential genes

The HCC dataset was accessed from the Gene Expression Omnibus (GEO) databank (https://www.ncbi.nlm.nih.gov/geo), and the species was human. Data were analyzed using the limma software package (Walter and Eliza Hall Institute of Medical Research, Melbourne, Australia) for differential analysis to identify differentially expressed genes in HCC, conditional on |logFC|>1 and corrected *P* < .05.

### 2.3. Acquisition of compounds and targets of action of drug GHHGG

Traditional Chinese Medicine Systems Pharmacology Database and Analysis Platform (TCMSP)^[11]^ (https://tcmsp-e.com/tcmsp.php) was used to analyze the data. The compounds were searched using the keywords of each constituent Chinese medicine of GHHGG. To obtain the effective combinations of GHHGG and their corresponding action targets, the screening conditions were established with a requirement of oral bioavailability of at least 30% and pharmacophore drug-likeness of at least 0.18. For Chinese medicines not searched in the TCMSP platform, high-throughput Chinese medicine experiments and reference databases, such as the HERB (http://herb.ac.cn/v2) database,^[12]^ were used to identify the active ingredients. The information obtained on the active compounds of GHHGG corresponding to the targets of action was converted into protein names and gene names with the assistance of UniProt (Universal Protein, https://www.uniprot.org/), and the standardized terms of the targets of action were obtained. The structural formulae of the GHHGG components were adapted from the PubChem database (https://pubchem.ncbi.nlm.nih.gov/) to predict the corresponding targets by applying the SwissTargetPrediction Tool (Swiss Institute of Bioinformatics, Lausanne, Switzerland, http://www.swisstargetprediction.ch/). To ensure that no ingredients or targets were missed, multiple databases were utilized.

We obtained core targets of GHHGG and liver cancer by intersecting liver cancer targets from Section 2.1, HCC differential targets from Section 2.2, and drug targets from Section 2.3.

### 2.4. Establishment of the protein-protein interaction (PPI) network

A PPI network was established using Search Tool for the Retrieval of Interacting Genes/Proteins (STRING; https://string-db.org/). To select polyproteins, we set the biological species to “Homo sapiens.” A PPI network was created to simplify connections and remove free targets. Predicted targets of GHHGG for the treatment of HCC were imported into Cytoscape (Cytoscape Consortium, San Diego) to study interactions between target genes. The resulting network nodes were analyzed using the CytoHubba plugin (National Yang-Ming University, Taipei, Taiwan) to calculate the degree values and build new networks according to the degree values.

### 2.5. Enrichment assay for Gene Ontology (GO) and the Kyoto Encyclopedia of Genes and Genomes (KEGG)

GO functional enrichment analysis of the potential targets was performed with categories covering biological process (BP), cellular component (CC), and molecular function (MF). KEGG pathway enrichment analysis was also conducted. Both analyses were carried out using the online bioinformatics analysis and visualization cloud platform^[13]^ (https://www.bioinformatics.com.cn/).

### 2.6. Survival analysis and differential analysis

Gene Expression Profiling Interactive Analysis 2 (GEPIA2; http://gepia2.cancer-pku.cn/) was utilized to perform survival and differential analyses. GEPIA2 data were acquired from The Cancer Genome Atlas and Genotype-Tissue Expression databases. In this study, the intersecting genes were entered into this platform to analyze their predictive values for overall survival (OS) and disease-free survival (DFS), and the targets with differences in both OS and DFS were used as critical targets.

### 2.7. Molecular docking

Molecular docking was performed between key targets with survival differences and the corresponding active ingredients. The 3-D structures of essential target proteins are available for download in the Protein Data Bank (https://www.rcsb.org/), and the 2-D chemical forms of the ligands were obtained from the TCMSP and PubChem databases (https://pubchem.ncbi.nlm.nih.gov/), respectively. The target proteins were presented as receptors, and nonpolar hydrogen was added to the water molecules. We utilized AutoDock (The Scripps Research Institute, La Jolla) and PyMOL (Schrödinger, Inc., New York) software to dock the active proteins and their components and visualize the results. The optimal docking conformation was determined on the basis of the lowest binding energy. If the binding energy was below −5 kJ/mol, the binding was considered spontaneous. Targets with docking points below −5 kJ/mol were deemed critical for further analysis.

## 3. Results

### 3.1. Liver cancer genes

To search for disease-related genes, we utilized the GeneCards, OMIM, and DisGeNET databases. The disease-related genes obtained from these databases were combined, and duplicated genes were deleted, resulting in 26,975 liver cancer-related genes.

### 3.2. Screening differentially expressed genes in HCC

**Figure 2.**
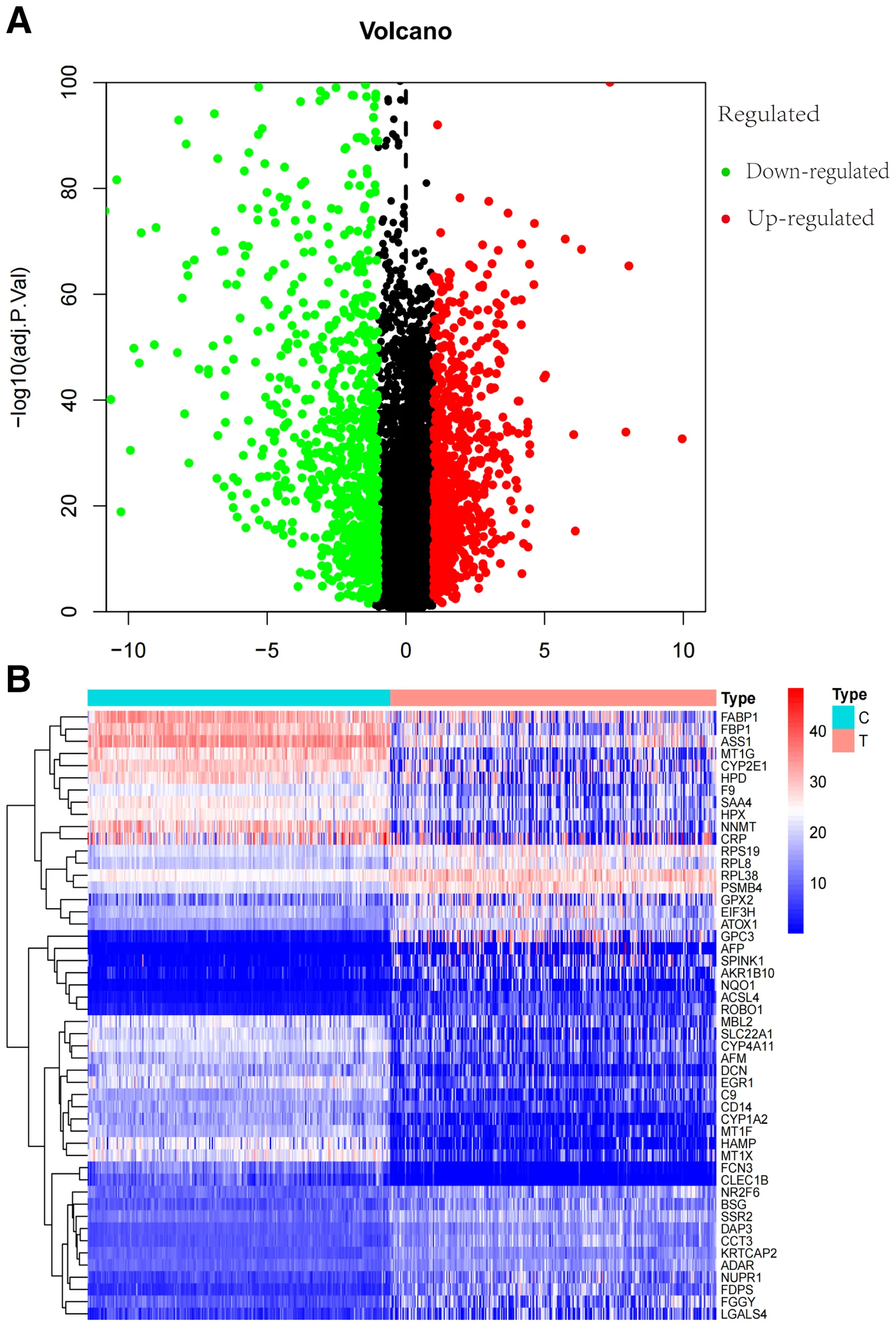
Differential gene analysis. (A) Volcanic map of differential; (B) heatmap of differential genes.

We downloaded the gene expression dataset GSE25097 (268 cases of HCC tissues, 243 instances of paracancerous tissues, and 5 cases of normal tissues) from the GEO database and identified differentially expressed genes (adjusted *P* < .05, |logFC|>1) involved in liver cancer lesions using the limma package, and the results are shown in Figure 2. Of these genes, 800 factors were upregulated, and 863 factors were downregulated.

### 3.3. Ingredients and related targets of GHHGG

The active ingredient targets of the drug were found in the TCMSP and HERB databases, and UniProt was used to realize protein-gene name conversion. On the other hand, we obtained the chemical structure formula of the ingredients from PubChem, imported them into SwissTargetPrediction to obtain the corresponding targets, combined the received data to remove duplicates, and finally obtained 152 active ingredients and 898 target genes.

**Figure 3.**
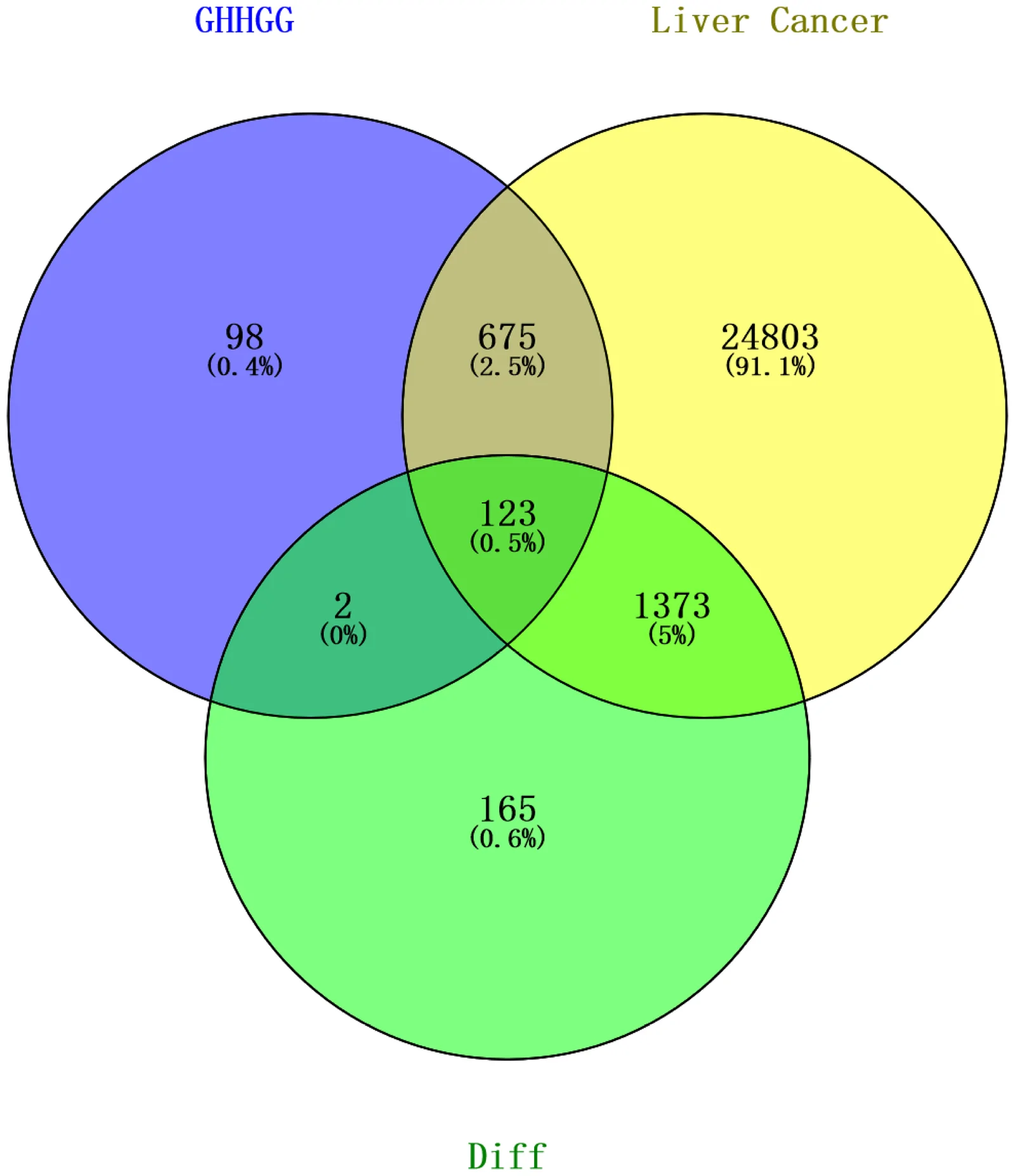
Venn diagram for GHHGG, liver cancer targets, and hepatocellular carcinoma differential targets. GHHGG = Gehua Hugan Gao.

The intersection of the liver cancer targets, HCC differential targets, and GHHGG drug targets was used to obtain 123 core targets (Fig. 3).

### 3.4. PPI network

**Figure 4.**
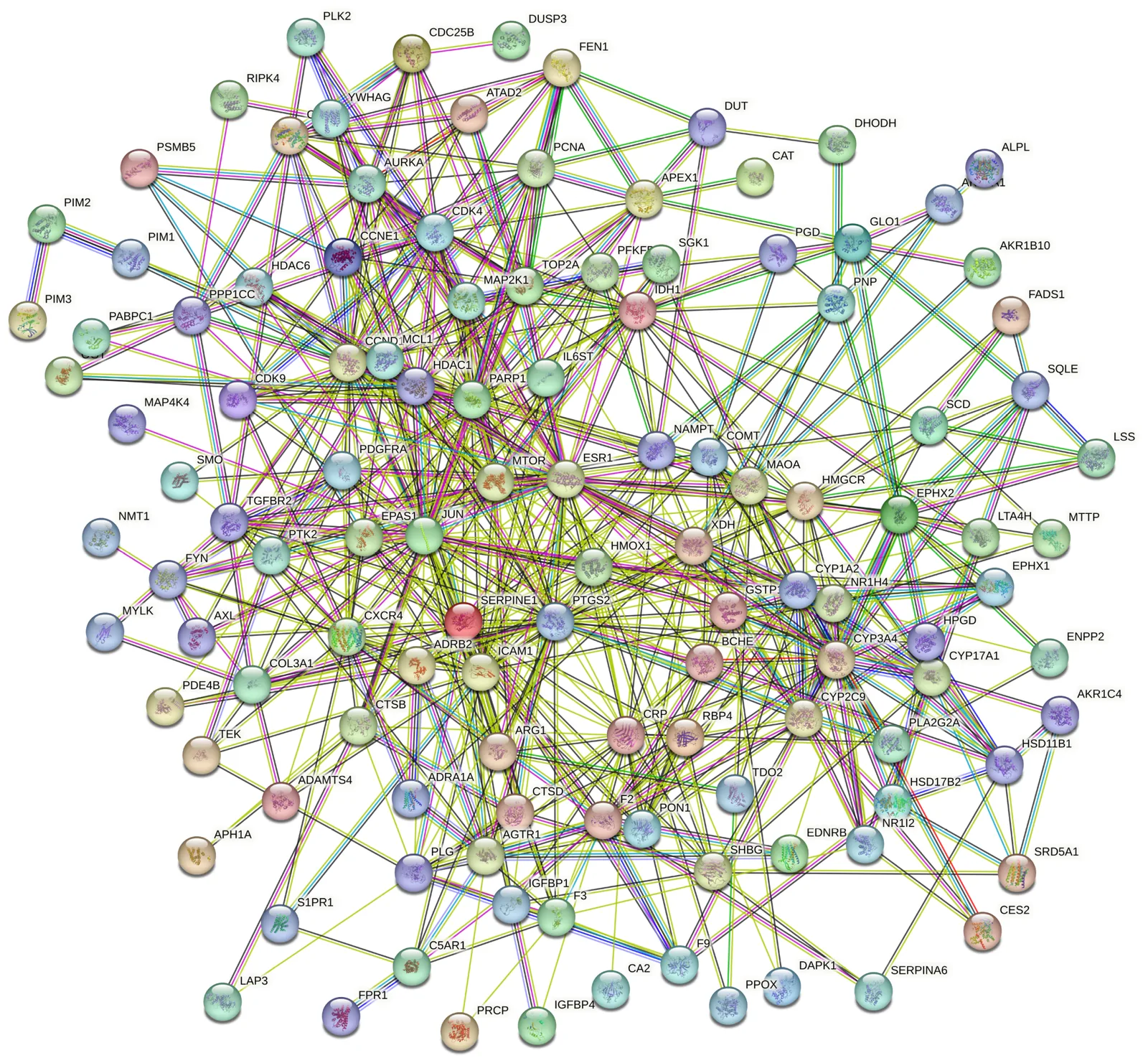
PPI network was constructed from 120 predicted targets. PPI = protein-protein interaction.

**Figure 5.**
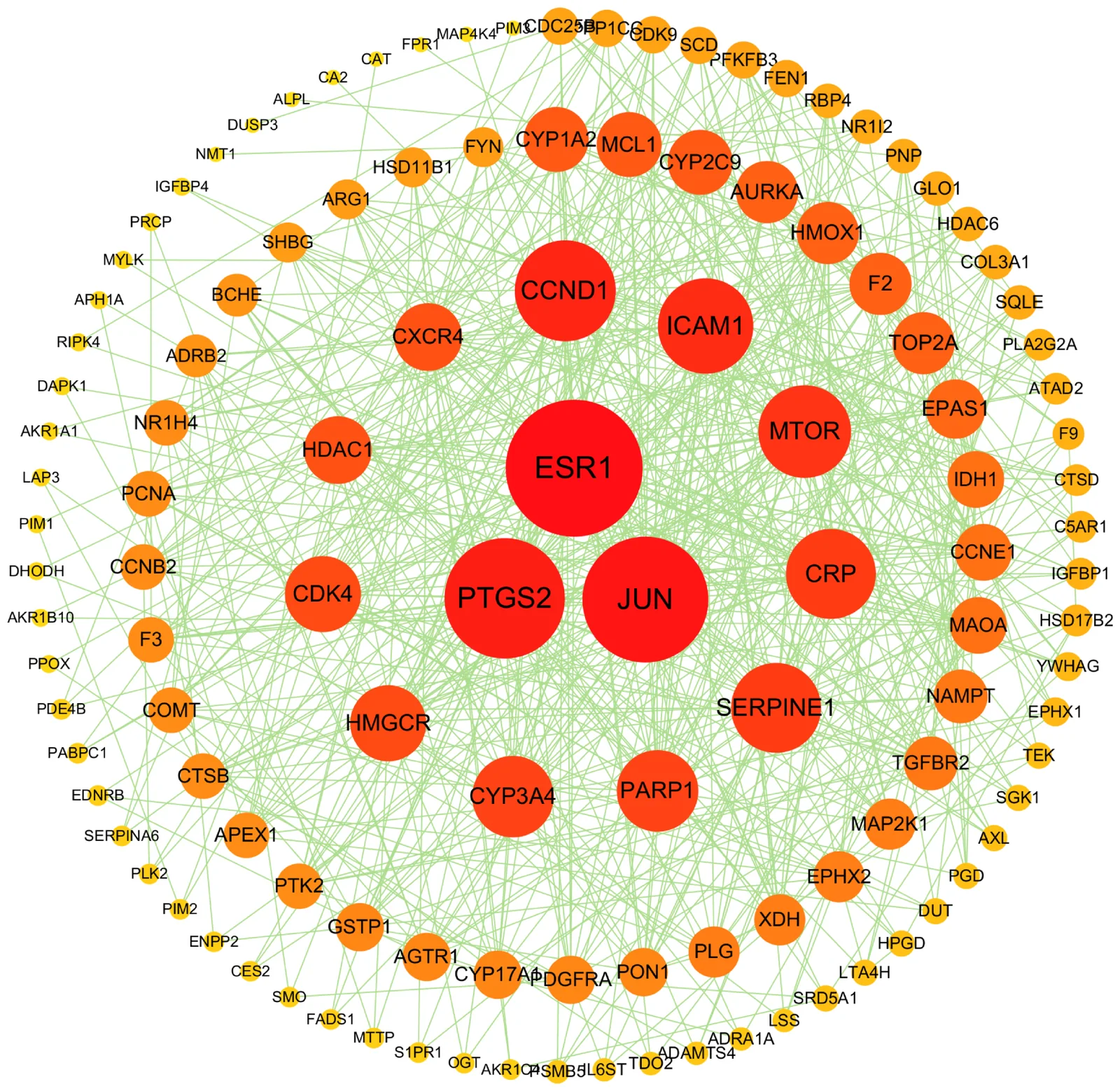
In the CytoHubba topological analysis, the red-to-yellow gradient indicates the degree value of the target in the PPI network. PPI = protein-protein interaction.

One hundred twenty-three intersecting targets were entered into the STRING (https://string-db.org/) database to further identify core targets. We selected “multiple proteins” and set “Homo sapiens” as the biological species. The PPI network contained 120 nodes, 610 edges, and a mean node degree of 10.2. Its enrichment *P*-value was <1.0e−16, and it was created to visualize complex connections and remove unrelated targets (Fig. 4). The CytoHubba plugin software was used within Cytoscape to perform the task. The nodal degree was calculated using the CytoHubba plugin, and a new PPI network was constructed in order of degree values (Fig. 5).

### 3.5. Enrichment assay for GO and KEGG

We analyzed the top 75 core targets with PPI degree values by GO and KEGG pathway enrichment via the online bioinformatics analysis and visualization cloud platform, Microbial Letter (https://www.bioinformatics.com.cn/).

**Figure 6.**
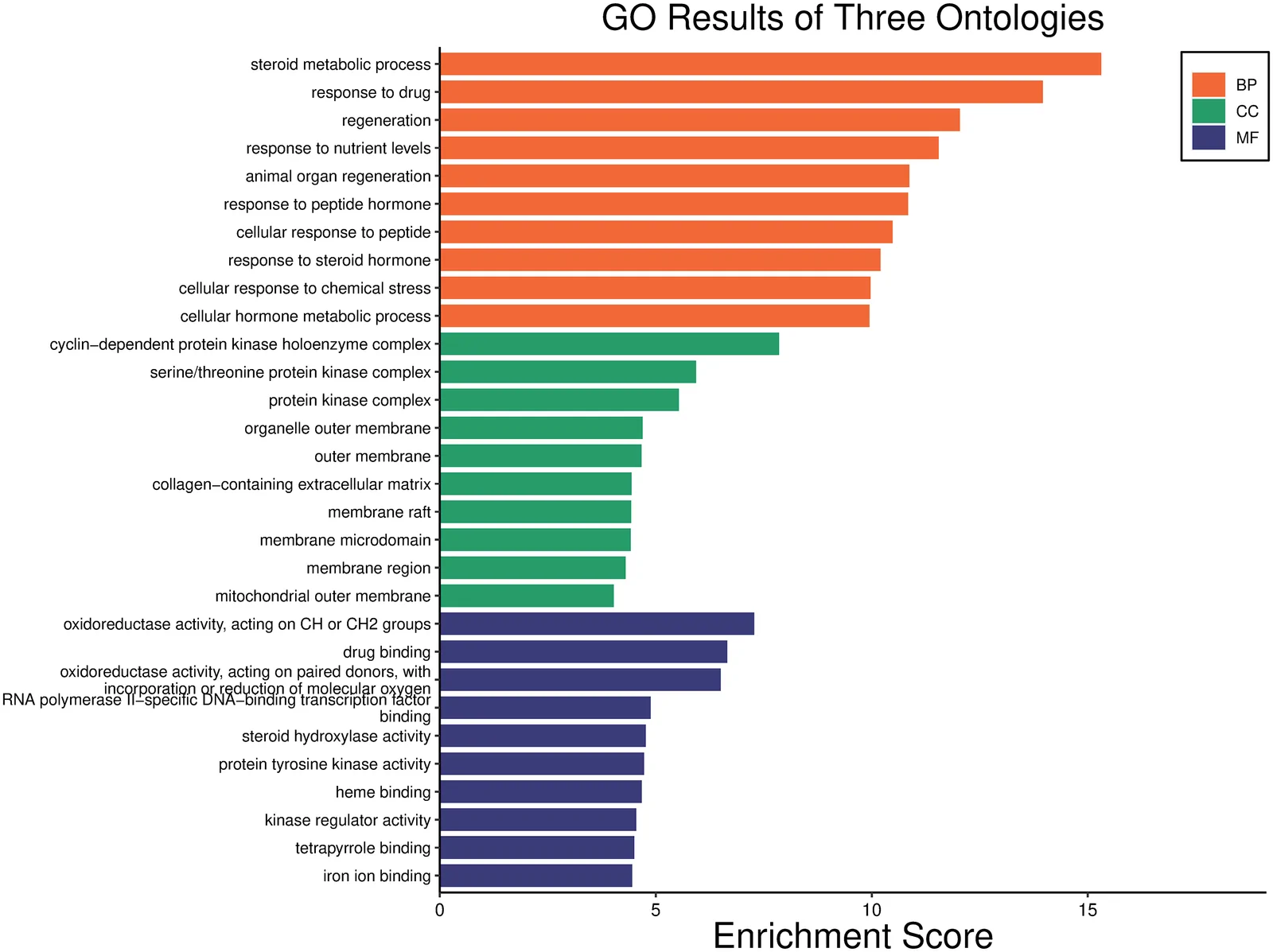
GO results for 3 ontologies. BP = biological process, CC = cellular component, GO = Gene Ontology, MF = molecular function.

We identified 813 GO functional enrichment entries that were statistically significant (*P* < .01). These entries included 702 BPs, 78 MFs, and 33 CCs. GO terms were sorted based on their *P*-values. We selected the top 10 terms in BP, CC, and MF, which were plotted in a column chart for easy visualization (Fig. 6). The results of the GO analysis suggest that GHHGG may play a role in BPs related to steroid metabolism and response to drugs, regeneration, animal organ regeneration, cellular response to peptides, and response to peptide hormones. MF predominantly consists of oxidoreductase activity, acting on methine/methylene groups, oxidoreductase activity, drug binding, acting on paired donors with incorporation or reduction of molecular oxygen, RNA polymerase II specific DNA binding transcription factor binding, and steroid hydroxylase activity. Regarding cellular composition, the main compounds are the cyclin-dependent protein kinase holoenzyme complex, organelle outer membrane, collagen-containing extracellular matrix, and membrane raft.

**Figure 7.**
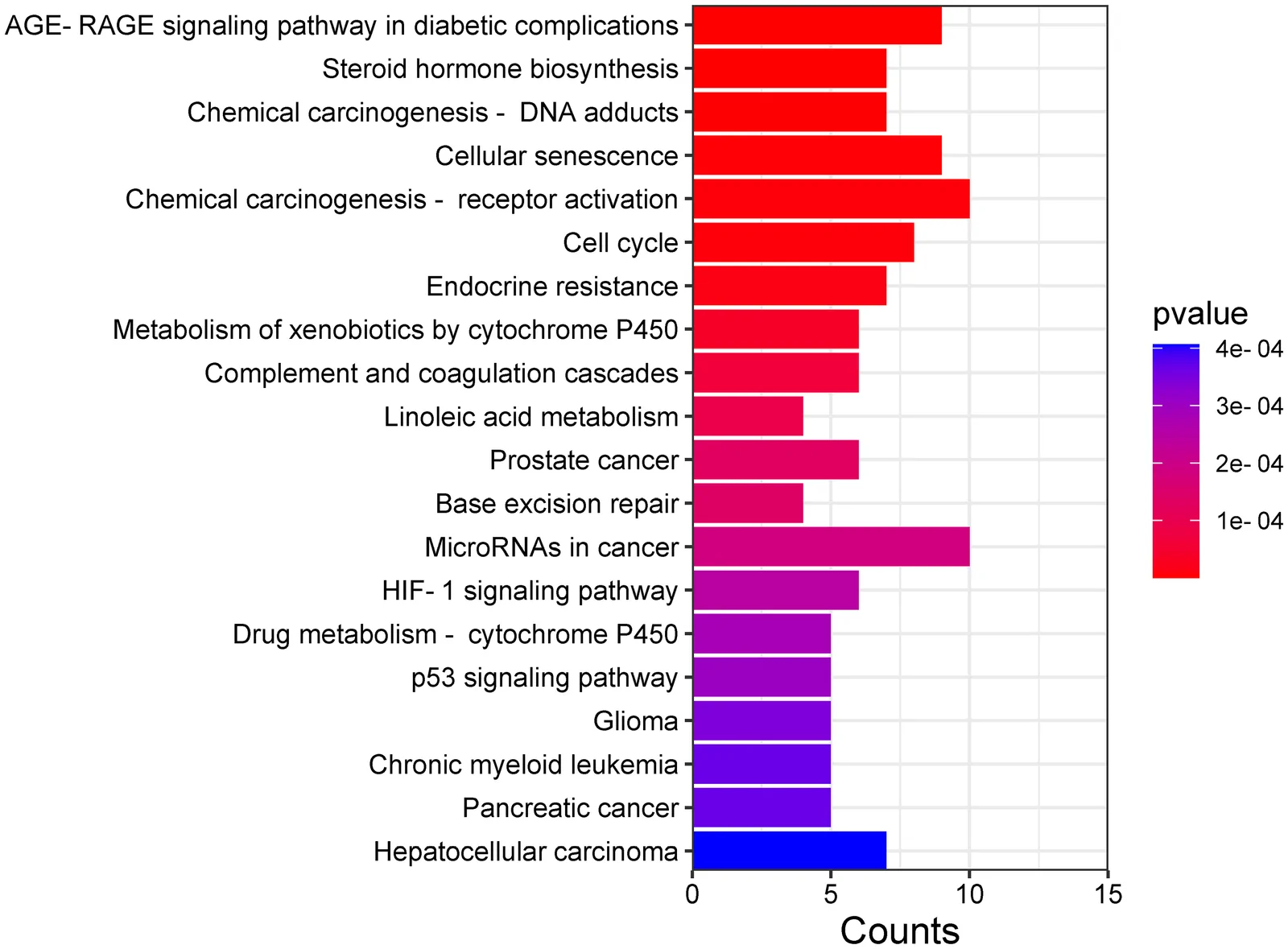
The top 20 KEGG pathways most enriched in GHHGG are ranked by *P*-value. AGE-RAGE = advanced glycation end-products–receptor for advanced glycation end-products, GHHGG = Gehua Hugan Gao, KEGG = Kyoto Encyclopedia of Genes and Genomes.

KEGG enrichment analysis was used to screen for 58 related pathways (*P* < .01). The bar chart represents 20 underlying signaling pathways sorted by *P*-values (Fig. 7). Bar length indicates the total number of enriched genes on the pathway. The bar color reflects the *P*-value size, with red bars indicating higher pathway significance. The 75 genes at the intersection were primarily associated with KEGG pathways, such as the hypoxia-inducible factor 1 (HIF-1) and P53 signaling pathways, chemical carcinogenesis-DNA induced, cellular senescence, chemical carcinogenesis-receptor activation, cell cycle, prostate cancer, microRNAs in cancer, glioma, pancreatic cancer, chronic myeloid leukemia, and HCC. The results show that GHHGG may modulate the HIF-1 and P53 signaling pathways to treat liver cancer by interfering with cellular senescence, the cell cycle, and angiogenesis.

### 3.6. Survival analysis and differential analysis

The predictive value of each critical gene for OS and DFS was analyzed using GEPIA2. Six critical targets with differences in OS and DFS were identified: estrogen receptor 1 (ESR1), cytochrome P450 3A4 (CYP3A4), cyclin-dependent kinase 4 (CDK4), type IIA topoisomerase (TOP2A), aurora kinase A (AURKA), and cyclin E1 (CCNE1).

**Figure 8.**
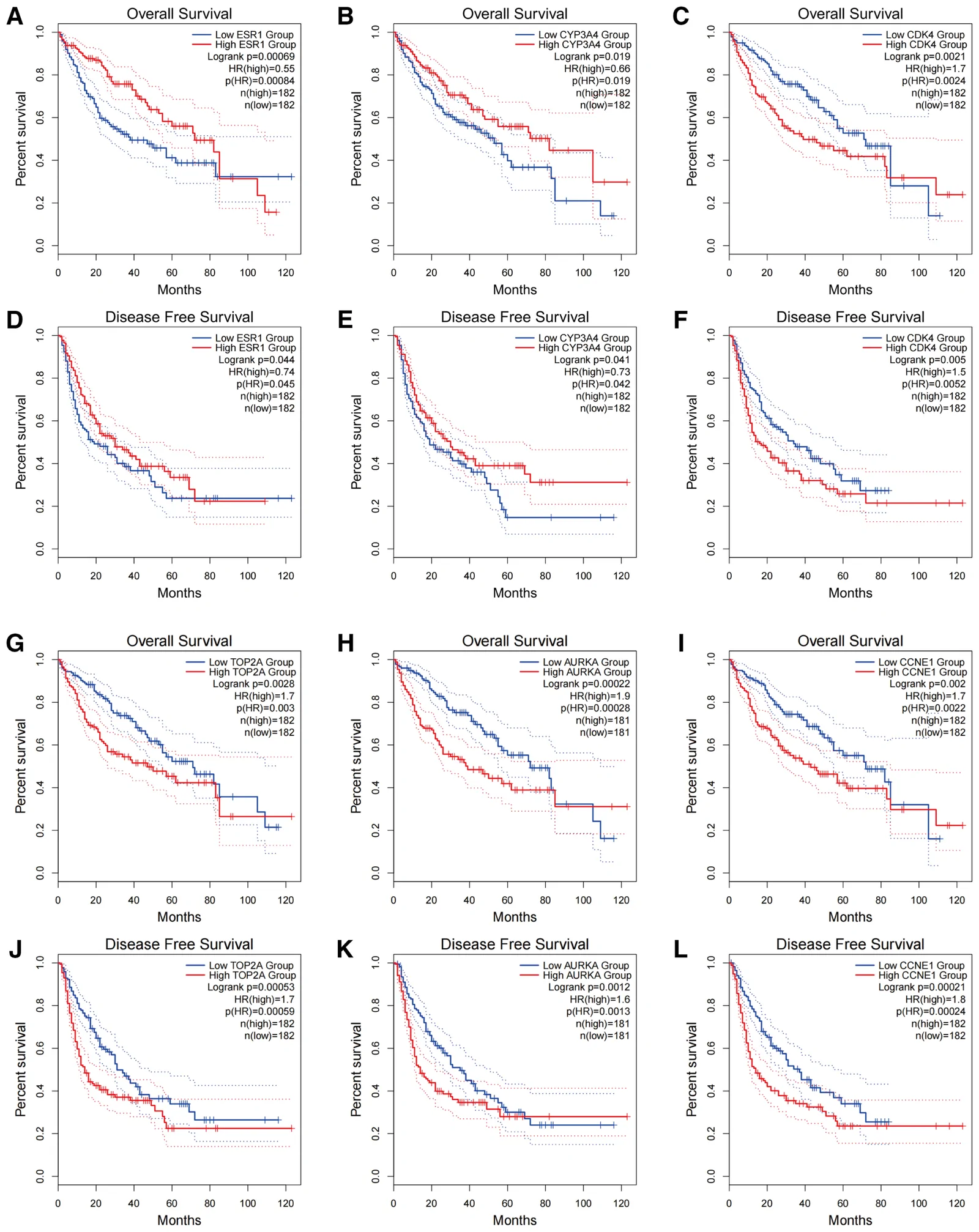
Survival analysis. (A) ESR1-OS; (B) CYP3A4-OS; (C) CDK4-OS; (D) ESR1-DFS; (E) CYP3A4-DFS; (F) CDK4-DFS; (G) TOP2A-OS; (H) AURKA-OS; (I) CCNE1-OS; (J) TOP2A-DFS; (K) AURKA-DFS; (L) CCNE1-DFS. AURKA = aurora kinase A, CCNE1 = cyclin E1, CDK4 = cyclin-dependent kinase 4, CYP3A4 = cytochrome P450 3A4, DFS = disease-free survival, ESR1 = estrogen receptor 1, HR = hazard ratio, OS = overall survival, TOP2A = type IIA topoisomerase.

**Figure 9.**
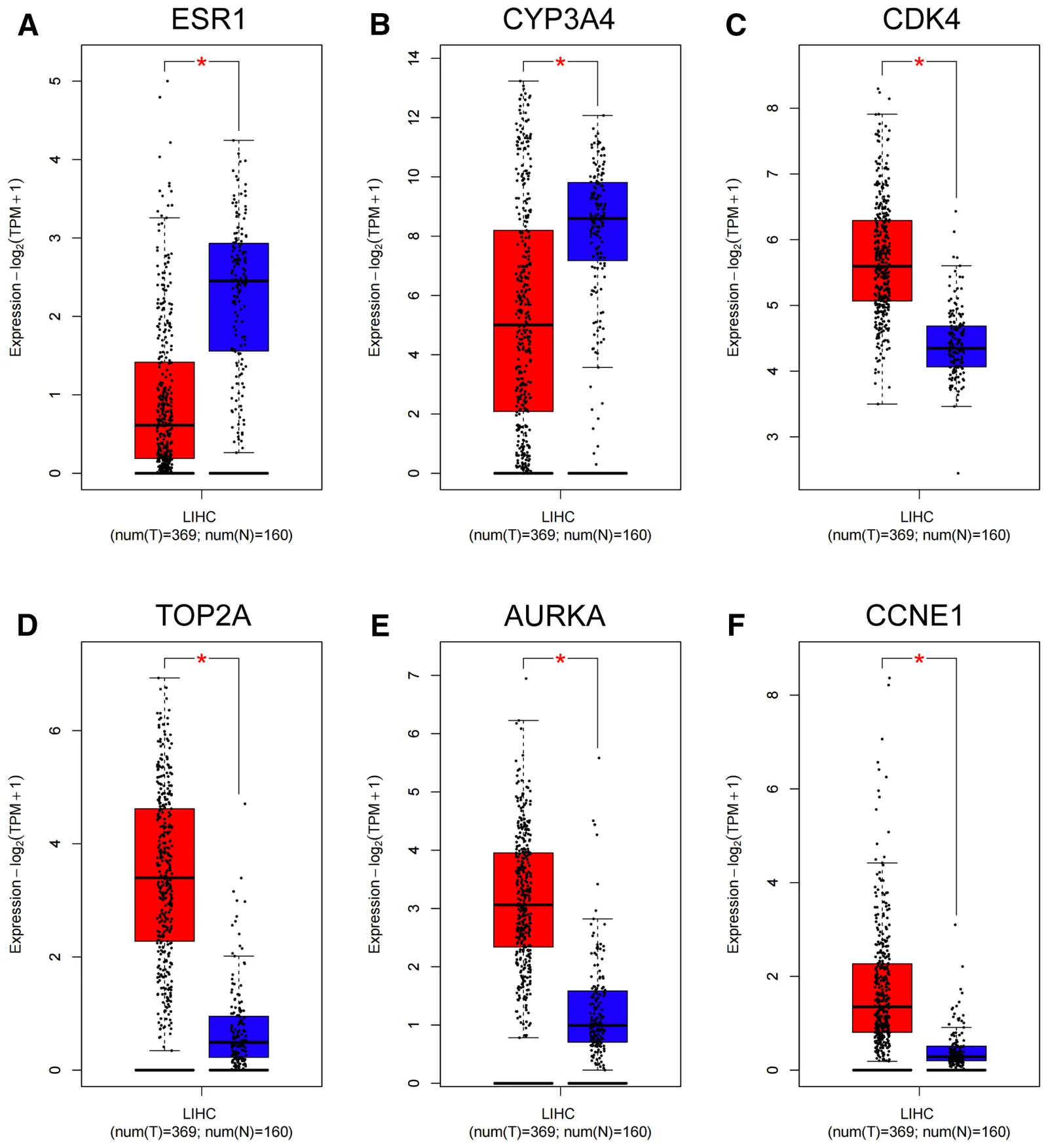
Differential expression analysis of essential genes. (A) ESR1; (B) CYP3A4; (C) CDK4; (D) TOP2A; (E) AURKA; (F) CCNE1. AURKA = aurora kinase A, CCNE1 = cyclin E1, CDK4 = cyclin-dependent kinase 4, CYP3A4 = cytochrome P450 3A4, ESR1 = estrogen receptor 1, LIHC = liver hepatocellular carcinoma, TOP2A = type IIA topoisomerase, TPM = transcripts per million.

Kaplan–Meier survival analysis and Cox proportional hazards regression were performed using GEPIA2 based on The Cancer Genome Atlas liver cancer dataset. Because GEPIA2 outputs only the hazard ratio (HR) and *P*-value, and does not provide numerical 95% confidence intervals, the results below and in Table 1 report only the HR and exact *P*-values. The 95% confidence intervals are visualized as dashed lines in the survival curves and are provided for reference. The analysis showed that high expression of ESR1 was significantly associated with longer OS (HR = 0.55, *P* < .001) and a similar protective trend for DFS (HR = 0.74, *P* = .045). High expression of CYP3A4 also indicated a favorable prognosis (OS: HR = 0.66, *P* = .019; DFS: HR = 0.73, *P* = .042). In contrast, elevated expression of CDK4, TOP2A, AURKA, and CCNE1 was significantly associated with poor prognosis (Table 1). Among them, high AURKA expression carried the highest risk of death (OS: HR = 1.90, *P* < .001), while high CCNE1 expression was most strongly associated with increased risk of recurrence (DFS: HR = 1.80, *P* < .001). Detailed statistics for all genes are provided in Table 1, and the corresponding survival curves are shown in Figure 8. The differential analysis results for these 6 key targets were analyzed for differences, and the results again verified that all 6 key targets differed (Fig. 9).

**Table 1:** Survival analysis results of the 6 key genes based on GEPIA2.

Gene	OS HR	OS *P*-value	DFS HR	DFS *P*-value
ESR1	0.55	.00084	0.74	.045
CYP3A4	0.66	.019	0.73	.042
CDK4	1.70	.0024	1.50	.0052
TOP2A	1.70	.003	1.70	.00059
AURKA	1.90	.00028	1.60	.0013
CCNE1	1.70	.0022	1.80	.00024

AURKA = aurora kinase A, CCNE1 = cyclin E1, CDK4 = cyclin-dependent kinase 4, CYP3A4 = cytochrome P450 3A4, DFS = disease-free survival, ESR1 = estrogen receptor 1, HR = hazard ratio, OS = overall survival, TOP2A = type IIA topoisomerase.

### 3.7. Molecular docking

**Figure 10.**
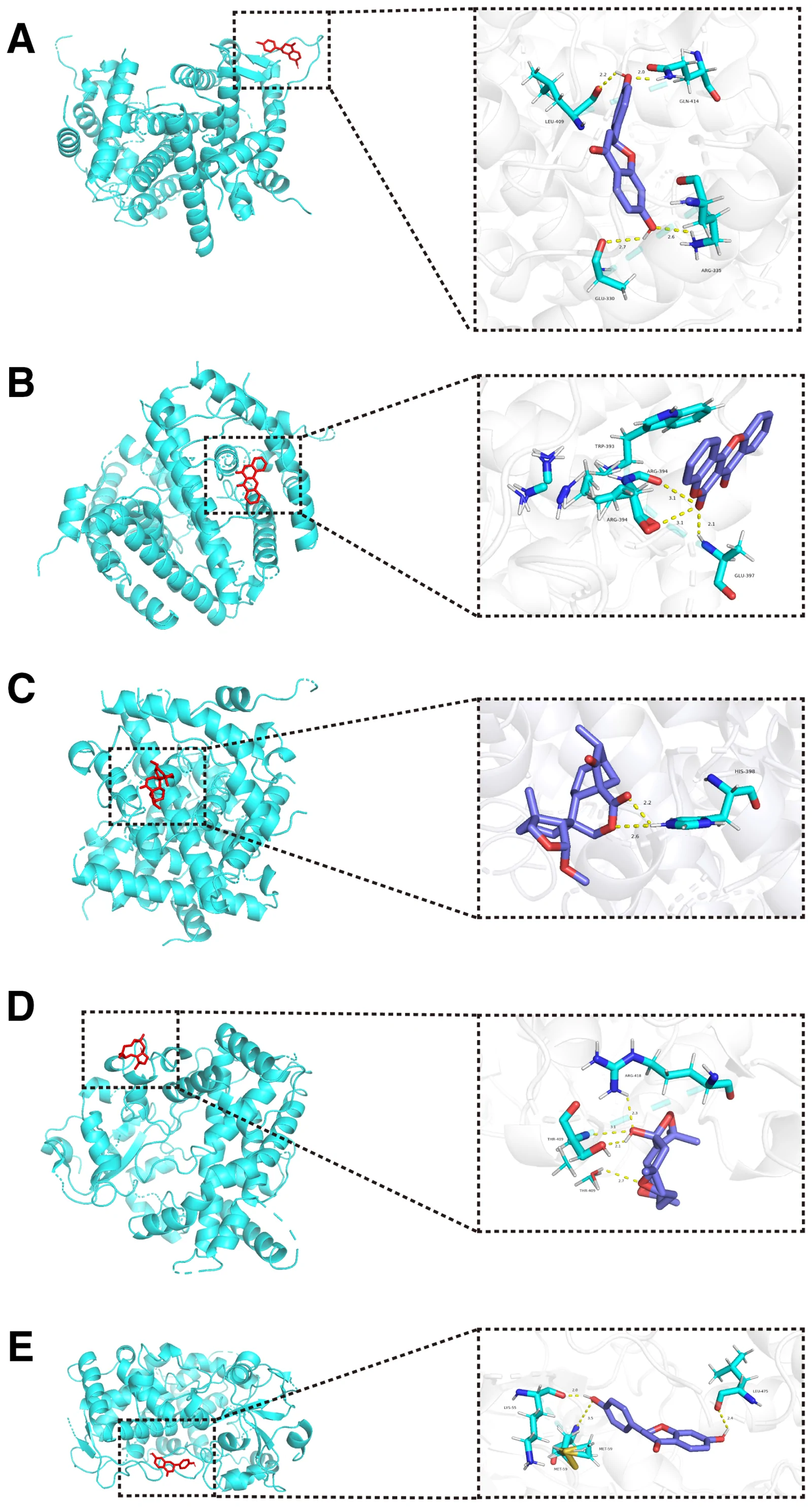
Molecular docking diagrams. (A) ESR1-DFV; (B) ESR1-Frutinone A; (C) ESR1-Ludongnin F; (D) CYP3A4-Wenjine; (E) CYP3A4-DFV. CYP3A4 = cytochrome P450 3A4, DFV = 7,4'-dihydroxyflavone, ESR1 = estrogen receptor 1.

We utilized the 6 core targets screened for molecular docking with the corresponding compounds. Ligands and receptors with binding energies not greater than −5 kcal/mol were screened (Table 2). The docking results indicated the effectiveness of GHHGG chemical compounds in binding to the core targets and thus functioning. Visual images of the first 2 core targets attached to the compound molecules are shown in Figure 10.

**Table 2:** Molecular docking results.

Target	Molecule	Binding
ESR1	DFV	−5.03
	Frutinone A	−5.19
	Ludongnin F	−5.52
CYP3A4	DFV	−5.15
	Wenjine	−5.06
CDK4	Inermin	−5.04
	Guidongnin B	−5.2
TOP2A	Wenjine	−5.25
AURKA	Celabenzine	−5.5
	Obscurine	−5.12
	Girinimbin	−6.58
	Ludongnin A	−5.03
	Guidongnin C	−5.32
CCNE1	Fumarine	−5.38

AURKA = aurora kinase A, CCNE1 = cyclin E1, CDK4 = cyclin-dependent kinase 4, CYP3A4 = cytochrome P450 3A4, DFV = 7,4'-dihydroxyflavone, ESR1 = estrogen receptor 1, TOP2A = type IIA topoisomerase.

## 4. Discussion

By 2025, liver cancer is expected to affect over 1 million people worldwide.^[3]^ Based on the theory of “Drinking Injury” and years of clinical experience in cancer prevention and treatment, Prof Yang Zhu, a Qihuang scholar in China, has prepared GHHGG based on the Gehua Jiecheng Decoction for resolving the effect of the drinker, which was recorded by Li Gao, a famous Chinese doctor of TCM in the Jin and Yuan Dynasties (1115–1234), in the *Treatise on the Spleen and Stomach*, and has added several TCMs that are commonly used in the treatment of tumors in the clinic to form the paste of TCM. In TCM, “paste” refers to a specific dosage form. It has many flavors of Chinese medication, is convenient to take, has a good taste, and has good patient compliance. GHHGG has been applied as an adjuvant therapy for liver cancer for many years at the First Affiliated Hospital of Guizhou University of TCM. The study analyzed active ingredients using network pharmacology, bioinformatics, and molecular docking to predict their mechanisms of action.

We identified 26,975 potential liver cancer targets using the DisGeNET, GeneCards, and OMIM databases and merged them into the “drug-target-disease” network. The liver cancer dataset GSE25097 was obtained from the GEO database, and the microarray data were analyzed using Perl (The Perl Foundation, https://www.perl.org/) and R software tools (R Foundation for Statistical Computing, Vienna, Austria). The limma package was used for differential analysis to identify 1663 differentially expressed genes in HCC. At the same time, 898 corresponding targets of GHHGG were obtained using TCMSP, HERB, UniProt, PubChem, and SwissTargetPrediction. The acquired liver cancer disease, differential, and drug targets were intersected to obtain 123 core targets. STRING was used to generate a PPI network, and the CytoHubba plugin was used to calculate the degree values of the active ingredients and corresponding marks. A new PPI network was created based on degree values in a specific order (Fig. 5). The enrichment of GHHGG targets for liver cancer treatment was explored using GO and KEGG analyses to investigate gene function and signaling pathways. These targets are involved in multiple signaling pathways, including multiple tumor signaling pathways, such as HIF-1 and P53 signaling pathways. Survival and differential analyses were performed via the GEPIA2 platform, and the genes with differences were used as critical targets for molecular docking with the corresponding active ingredients. The targets ESR1, CYP3A4, CDK4, TOP2A, AURKA, and CCNE1 were found to be closely related to hepatocarcinogenesis.

Survival and differential gene analyses revealed that ESR1 and CYP3A4 are expressed at low levels in HCC. Their high expression was favorable for overall survival and DFS. In contrast, CDK4, TOP2A, AURKA, and CCNE1 genes were highly expressed in HCC, and their high expression was unfavorable for OS and DFS. ESR1,^[14–16]^ CYP3A4,^[17]^ CDK4,^[18,19]^ TOP2A,^[14,15,20,21]^ AURKA,^[15,22,23]^ and CCNE1^[24,25]^ have been shown in many publications to be key biomarkers for HCC.

The genes associated with favorable prognosis and hormonal/metabolic protection were mainly ESR1 and CYP3A4. ESR1 encodes estrogen receptor alpha, and estrogen signaling is considered protective in HCC^[26]^; defective estrogen signaling may contribute to hepatocarcinogenesis, consistent with the 2- to 3-fold higher incidence and mortality of liver cancer in men.^[1]^ Wang et al demonstrated that inhibition of ESR1 expression promotes the dissemination of HCC cells.^[27]^ Interestingly, ligand-free ESR1 has been shown to regulate CYP3A4 expression in the human liver.^[28]^ CYP3A4, which is expressed in the liver and lungs,^[29]^ appears to exert tumor-suppressive effects: reduced CYP3A4 expression predicts early HCC recurrence and increased carcinoma risk,^[17]^ and CYP3A4 inhibits HCC cell proliferation by downregulating glycolytic flux.^[30]^ Taken together, these observations suggest that ESR1 and CYP3A4 predominantly confer protective and prognostically favorable effects in HCC, although ESR1 alone has limited diagnostic accuracy.^[15]^

The cell-cycle-related genes linked to poor prognosis were CDK4, TOP2A, AURKA, and CCNE1. In contrast, CDK4, TOP2A, AURKA, and CCNE1 are intimately involved in cell-cycle regulation, and their upregulation is closely associated with unfavorable outcomes in HCC. CDK4 is a key driver of the G1–S phase transition and promotes cell proliferation in various malignancies.^[18,31]^ TOP2A enhances HCC invasion, migration, and epithelial–mesenchymal transition, and its overexpression positively correlates with poor prognosis^[32]^; silencing of TOP2A can also reverse acquired resistance to regorafenib.^[33]^ AURKA, a classical cell-cycle-associated kinase,^[34]^ is required for the invasive and migratory capacity of HCC cells, and AURKA inhibitors have been shown to reduce proliferation, migration, and invasion while promoting apoptosis, highlighting their therapeutic potential for advanced HCC.^[35–37]^ Similarly, CCNE1 is a critical regulator of HCC progression: its overexpression drives hepatocarcinogenesis and chromosomal instability, and therapeutic inhibition or genetic ablation of CCNE1 significantly reduces tumor burden in mouse models.^[24,38–40]^ Notably, while ESR1 has limited diagnostic utility, both AURKA and TOP2A have been proposed as diagnostic biomarkers for HCC,^[15]^ further supporting the clinical relevance of this gene cluster.

The survival analysis represents one of the most clinically informative aspects of this study, as it directly links gene expression patterns to patient prognosis. Our results demonstrate that high expression of ESR1 and CYP3A4 is significantly associated with longer OS and DFS, with ESR1 showing a particularly strong protective effect on OS (HR = 0.55, *P* < .001). These findings suggest that ESR1 and CYP3A4 may serve as favorable prognostic biomarkers in HCC, and their expression levels could potentially be used to identify a subgroup of patients with a more indolent disease course who may benefit from less intensive surveillance or tailored treatment strategies. Conversely, elevated expression of CDK4, TOP2A, AURKA, and CCNE1 was robustly associated with shorter OS and DFS. Among these, AURKA conferred the highest risk of death (OS: HR = 1.90, *P* < .001), while CCNE1 was most strongly linked to an increased risk of recurrence (DFS: HR = 1.80, *P* < .001). These cell-cycle-related genes not only highlight biologically aggressive tumor subtypes but also carry clear clinical implications: their overexpression could be used to stratify patients at high risk of recurrence and mortality, thereby guiding more aggressive adjuvant therapy or closer postoperative follow-up. Furthermore, several of these genes, in particular AURKA and TOP2A, are already being explored as therapeutic targets, and their prognostic significance lends additional support to the development of targeted inhibitors in HCC. Taken together, the integration of gene expression data with survival outcomes provides a clinically relevant framework for risk stratification and personalized management of liver cancer patients.

However, our study has some limitations. Some of the potential protein targets in the constituents of GHHGG were omitted because they were not found in existing liver cancer-related databases. It is imperative to further validate the intricate molecular mechanism of GHHGG to provide a reference for developing new biologics for experiments and clinical applications.

## 5. Conclusion

In summary, this study preliminarily identified, on the basis of network pharmacology, bioinformatics, and molecular docking, the mechanisms of the effects of GHHGG in treating liver cancer through crucial targets. Its drug-target-disease network relationship was revealed, and its potential effect mechanisms for treating liver cancer were explored. The results confirmed that GHHGG plays a role in treating liver cancer through integrated poly-component, poly-targeting, and poly-pathway effects. This provides promising evidence for the further experimental validation of these critical targets.

## Author contributions

**Conceptualization:** Zhu Yang, Bing Yang, Dongxin Tang.

**Data curation:** Guangze Ye, Dalin Chen, Dan Li.

**Formal analysis:** Guangze Ye, Hongting Yan.

**Funding acquisition:** Bing Yang, Fengxi Long, Wenyu Wu, Dongxin Tang.

**Methodology:** Hongting Yan, Hanbo Qiu.

**Investigation:** Bing Yang, Dan Li.

**Project administration:** Bing Yang.

**Resources:** Zhu Yang, Bing Yang.

**Software:** Guangze Ye, Dalin Chen, Hanbo Qiu.

**Supervision:** Bing Yang, Dongxin Tang.

**Validation:** Dalin Chen, Hongting Yan.

**Visualization:** Guangze Ye, Dalin Chen.

**Writing – original draft:** Guangze Ye.

**Writing – review & editing:** Guangze Ye, Bing Yang, Dongxin Tang.
